# Low-Frequency Bandgaps of the Lightweight Single-Phase Acoustic Metamaterials with Locally Resonant Archimedean Spirals

**DOI:** 10.3390/ma15010373

**Published:** 2022-01-05

**Authors:** Haoqiang Gao, Qun Yan, Xusheng Liu, Ying Zhang, Yongtao Sun, Qian Ding, Liang Wang, Jinxin Xu, Hao Yan

**Affiliations:** 1 Department of Mechanics and Tianjin Key Laboratory of Nonlinear Dynamics and Control, Tianjin University, Tianjin 300350, China; hqgao@tju.edu.cn (H.G.); liuxusheng0920@tju.edu.cn (X.L.); zy981490991@163.com (Y.Z.); liangwang9528@163.com (L.W.); 2 Key Laboratory of Aeroacoustics and Dynamics, Aircraft Strength Research Institute, Xi’an 710065, China; qunyan_ac@163.com (Q.Y.); hedyyh0912@163.com (H.Y.); 3 School of Civil Engineering, Henan Polytechnic University, Jiaozuo 454003, China; jinxin0312@outlook.com

**Keywords:** local resonant Archimedean spirals, low-frequency bandgaps, single-phase acoustic metamaterials, vibration attenuation

## Abstract

In order to achieve the dual needs of single-phase vibration reduction and lightweight, a square honeycomb acoustic metamaterials with local resonant Archimedean spirals (SHAMLRAS) is proposed. The independent geometry parameters of SHAMLRAS structures are acquired by changing the spiral control equation. The mechanism of low-frequency bandgap generation and the directional attenuation mechanism of in-plane elastic waves are both explored through mode shapes, dispersion surfaces, and group velocities. Meanwhile, the effect of the spiral arrangement and the adjustment of the equation parameters on the width and position of the low-frequency bandgap are discussed separately. In addition, a rational period design of the SHAMLRAS plate structure is used to analyze the filtering performance with transmission loss experiments and numerical simulations. The results show that the design of acoustic metamaterials with multiple Archimedean spirals has good local resonance properties, and forms multiple low-frequency bandgaps below 500 Hz by reasonable parameter control. The spectrograms calculated from the excitation and response data of acceleration sensors are found to be in good agreement with the band structure. The work provides effective design ideas and a low-cost solution for low-frequency noise and vibration control in the aeronautic and astronautic industries.

## 1. Introduction

Lightweight periodic honeycomb materials (LPHM) are a typical kind of acoustic metamaterials [1,2,3,4,5,6]. With the rational design of sub-wavelength units to control elastic wave propagation, these structures can be widely used in vibration isolation [7,8], noise insulators/absorbers [9,10], negative refraction [11,12], and acoustic cloaking [13,14]. As the Bragg scattering bandgap requires the lattice size to be comparable to the corresponding wavelength [15,16,17], the reduction of low-frequency vibrations and noise below 500 Hz remains a challenging assignment for LPHM design [18,19,20] when used in engineering applications in aeronautics [21] and railways [22].

Introducing a local resonant mass block is currently one of the popular ways to achieve a low-frequency bandgap below 500 Hz for LPHM. Liu et al. [23] used a simple cubic lattice form to constitute a local resonance unit using a high-density mass block wrapped in soft rubber material. The artificial periodic structure is formed by periodically arranged local resonance units in an elastic medium, which successfully exploits the local resonance effect of elastic waves to achieve a bandgap of around 400 Hz in a 20 mm cubic lattice. In contrast, traditional local resonance structures typically allow for a very narrow resonance bandgap at low frequencies [24]. For the purpose of obtaining a wide resonant bandgap, Dong et al. [25] proposed a new multiple bandgap phononic crystals capable of generating lots of flat bandgaps in the low-frequency band, however, the flat bandgaps generated by this unique local resonant phononic crystal are still all above 500 Hz. To address the above problem, Li et al. [26] proposed a phonon crystal structure in the form of a cylinder with a periodic arrangement attached to a thin plate. This cylinder structure attached to a thin plate corresponds to the formation of a “solid-state Helmholtz resonator” and succeeds in generating a large number of broadband gaps in the range 256 Hz–855 Hz. In addition, Ning et al. [27] similarly designed a tunable metamaterial consisting of a frame structure, airbag, and a counterweight by introducing a local resonant mass block. They modulated the designed acoustic metamaterials by the gauge pressure and gas temperature inside the airbag, effectively attenuating wave propagation in the 13 Hz to 90 Hz range. Although the low-frequency bandgap of LPHM below 500 Hz can be obtained by introducing local resonance mass blocks in the above-mentioned references, the lightweight efficiency of LPHM has severely deteriorated. On the other hand, the discontinuous distribution of materials with different properties in the complex spatial structure can bring great challenges to the fabrication [28,29].

To endow the LPHM with the simultaneous advantages of lightweight efficiency and low-frequency bandgaps, the single-phase lightweight periodic honeycomb materials with subwavelength bandgaps have attracted much attention in the research community [18,30,31,32,33,34]. Chen et al. [35,36] designed a star-assisted metamaterial with a low-frequency bandgap and double-negative characteristics in a certain frequency range using single-phase materials, which solved the problem that conventional double-negative acoustic metamaterials are difficult to be applied due to their complex structure and multi-phase material composition. The novel lightweight bidirectional re-entrant lattice metamaterials were proposed by Ren et al. [37], which forms a wide bandgap of about 2 kHz in the range of 2.7 kHz to 4.7 kHz. The single-phase acoustic metamaterials with periodically arranged diverging star-shaped cells resulting in a “low frequency” bandgap from 1.44 kHz to 1.56 kHz were proposed by Kumar and Pal [38]. In the above-mentioned, single-phase LPHM, although the simultaneous advantages of lightweight efficiency and low-frequency bandgaps are realized, the low-frequency bandgaps are all larger than 500 Hz.

Aiming at the challenging problem of vibration and noise reduction below 500 Hz for the single-phase LPHM, a new kind of local resonance single-phase lightweight periodic honeycomb materials, the square honeycomb acoustic metamaterials with locally resonant Archimedean spirals (SHAMLRAS) are proposed in this paper. The SHAMLRAS consists of Archimedean spirals of the same material combined in a square honeycomb structure. These acoustic metamaterials with the introduction of Archimedean spirals have special resonance characteristics compared to existing single-phase acoustic metamaterials and have the capacity to form multiple bandgaps below 500 Hz (down to approximately 184.5 Hz) within a lattice size of 40 mm. The research has the potential to provide an invaluable guide to the engineering suppression of low-frequency noise and vibration.

The paper is organized into six sections including the introduction above. The second part describes the geometry of SHAMLRAS and illustrates the tools for wave propagation studies. The low-frequency bandgap characteristics and directional attenuation properties of elastic waves in the SHAMLRAS periodic structure are thoroughly discussed in Section 3. For more flexibility in obtaining the low-frequency bandgap width we need, the effect of the material parameters, the arrangement of spirals, and the variables of parameter control equations on the bandgap width and position being investigated in Section 4. The transmission loss experiments and COMSOL verification of the low-frequency bandgap are demonstrated in Section 5. Finally, the major achievements of this work are presented in Section 6.

## 2. Mechanical Design and FE Modeling of the SHAMLRAS

### 2.1. Mechanical Design

**Figure 1:**
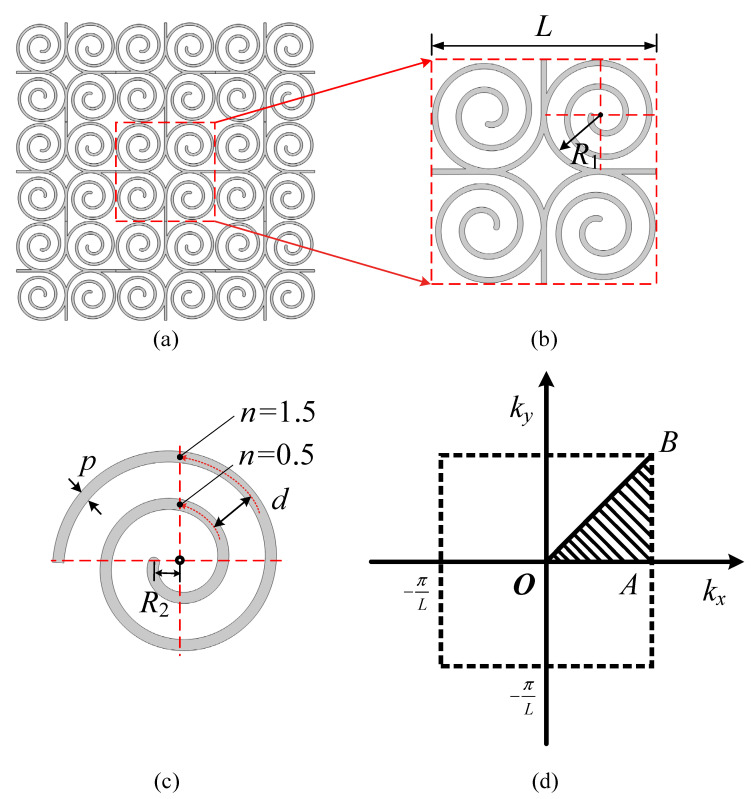
(**a**) Description of the square honeycomb acoustic metamaterials with locally resonant Archimedean spirals (SHAMLRAS); (**b**) the unit cell of SHAMLRAS; (**c**) the circular array unit of Archimedes spiral; (**d**) the first Brillouin zone and irreducible Brillouin zone (IBZ, black shadow region).

In this study, a low-frequency single-phase acoustic metamaterials consisting of multiple spirals is proposed, based on a square honeycomb structure and a circular array of four spirals. The structural composition and lattice arrangement of acoustic metamaterials are shown in Figure 1a–c, with a lattice constant of *L* = 40 mm and a ligament thickness of *p* = 1 mm. We can effectively reduce the bandgap frequency by tuning the parameters of the spiral line equation.

The spiral line control equation is derived from the Archimedean spiral line and is derived as follows:

(1) r=α+βθ

where *θ* is the pole angle, *α* is the pole diameter when *θ* = 0, i.e., the initial radius, and *β* is the increase (or decrease) of the pole diameter per 1 rad of rotation.

Suppose *R*_2_ is the inner diameter, *n* is the turn number, and *d* is the circle distance, then this polar equation can be written in the following form:

(2) $$\left\{ \begin{array}{l} r=\left(R_{2}+ndt\right)\\ \theta =n\cdot 2\pi t \end{array}\right.,\;t\in \left[0,1\right]$$

Therefore, the corresponding Cartesian coordinate equation for the Archimedean spiral plane is shown below, which is the parametric control equation for the spiral in this paper.

(3) $$\left\{ \begin{array}{l} x\left(t\right)=\left(R_{2}+ndt\right)\cos\left(n\cdot 2\pi t\right)\\ y\left(t\right)=\left(R_{2}+ndt\right)\sin\left(n\cdot 2\pi t\right) \end{array}\right.,\;t\in \left[0,1\right]$$

### 2.2. FE Modeling for the Free Wave Propagation

The unit cell and Brillouin zone of periodic lattices are shown in Figure 1d. In the unit, $e_{i}$ (i=1,2) is the basic lattice vector, which can be expressed by orthogonal Cartesian basic vector and lattice constant as:

(4) $$\begin{array}{l} e_{1}=Li\\ e_{2}=Lj \end{array}$$

A reciprocal lattice space is defined according to the direct lattice space. In general terms, the relationship between the basis vectors of the direct and reciprocal lattices is satisfied as follows:

(5) $$e_{i}\cdot e_{j}^{*}=2\pi \delta_{ij}$$

where $e_{i}$ denote the basis vectors of the direct lattice, $e_{j}^{*}$ denote the basis of reciprocal lattice, and the subscripts *i* and *j* take the integer value 1 and 2. The $\delta_{ij}$ is the Kronecker delta function, and the expressions are as follows:

(6) $$\delta_{ij}=\left\{ \begin{array}{l} 1,i=j\\ 0,i\neq j \end{array}\right.$$

The coordinate positions of the lattice points in the reciprocal lattice can be represented by the reciprocal lattice vector G, which is a linear combination of the reciprocal lattice basis vectors:

(7) $$G=r_{o}+n_{1}e_{1}+n_{2}e_{2}$$

where $n_{1}$ and $n_{2}$ are integers, and $r_{o}$ is the displacement of Point O.

Additionally, the reciprocal lattice vectors of the SHAMLRAS can be expressed as:

(8) $$\begin{array}{l} e_{1}^{*}=\left(\frac{2\pi }{L},0\right)\\ e_{2}^{*}=\left(0,\frac{2\pi }{L}\right) \end{array}$$

Thus, the entire SHAMLRAS periodic structure can be constructed by shifting the unit cell along the basic lattice vector ($e_{1}$,$e_{2}$) in a two-dimensional space. The Brillouin zone of the basic lattice can also be obtained, as shown in Figure 1d, in which the black shadow region is the irreducible Brillouin zone (IBZ). The coordinates of the boundary points of the IBZ are shown in Table 1.

According to Bloch’s theorem, the part u(r) of the eigenwave field volume associated with the spatial position r can be expressed in the form of a spatial plane wave

(9) $$u(r)=U_{k}(r)e^{-ik\cdot r}$$

where r=(x,y,z) is the position vector, $k=(k_{x},k_{y},k_{z})$ is the wave vector in the first Brillouin zone, *i* is the imaginary unit, and $U_{k}(r)$ is eigenwave amplitude.

The boundary displacement of the periodic structure can be controlled by Bloch boundary conditions (Floquet periodicity), so there is

(10) $$u(r+R)=e^{-ik\cdot r}u(r)$$

where r is the position vector of the node on the boundary, and R is the basis vector of the lattice.

Determine the form function according to the general finite element procedure and establish the stiffness and mass matrices within the unit to obtain the generalized eigenequations of the unit:

(11) $$\left(k_{s}-\omega^{2}M_{s}\right)u\left(\nu \right)=f$$

where $k_{s}$ is the unit stiffness matrix, $M_{s}$ is the mass matrix, u(ν) and f are the vectors of generalized nodal displacements and forces. 

The SHAMLRAS periodic structure can be reduced to a series of cells using Equation (10) for the whole calculation. Two edges in *x*- and *y*-directions are selected as source boundaries in COMSOL Multiphysics 5.5, and the two edges selected for the destination boundary correspond to the two edges of the source boundary. The Floquet periodicity conditions at the corresponding boundaries of the periodicity cell are expressed as:

(12) $$u_{\mathrm{destination}}=e^{-ik_{f}\left(r_{\mathrm{destination}}-r_{\mathrm{source}}\right)}u_{\mathrm{source}}$$

where u is a vector of dependent variables, and the vector $k_{f}$ represents the spatial periodicity of the excitation.

The wave vector k is parametrically swept along the path O→A→B→O according to the first Brillouin zone of the SHAMLRAS structure given in Figure 1d. With Equations (11) and (12), the structural eigenfrequencies can be found for a given k. Thus, the dispersion curve is finally plotted with the wave vector k as the horizontal coordinate and the eigenfrequency as the vertical coordinate to obtain the energy band diagram of the SHAMLRAS, and the forbidden band between the dispersion curves is the band structure. Moreover, the eigenfrequencies on the dispersion branches all correspond to the mode shape the structure has. The complete surface ω=ω(k) is the dispersion surface mentioned in Section 3.2. In this case, the corresponding dispersion surfaces of the different dispersion branches represent a class of modes at the corresponding eigenfrequencies. Hence, the *n*-th (n=1,2,3,…) dispersion surface can also be called the *n*-th (n=1,2,3,…) mode. In this case, there are as many orders of eigenfrequency as there are dispersion surfaces.

## 3. Bandgap and Directional Propagation Characteristics of Elastic Waves for the SHAMLRAS

### 3.1. Bandgap Characteristics

In this section, the in-plane dynamical properties of SHAMLRAS based on Figure 1 are investigated. The geometrical parameters of the SHMLRAS and the parameters of the photosensitive resin used are shown in Table 2.

**Figure 2:**
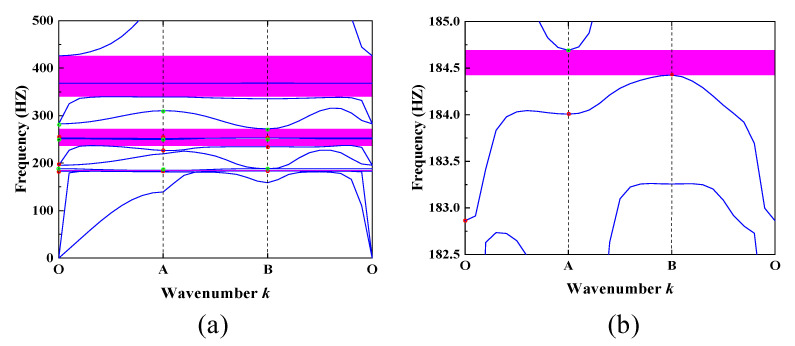
(**a**) Band structure of the SHMLRAS and (**b**) detailed schematic of the first bandgap.

Based on the material and geometrical parameters provided, the calculated bandgap structure of the SHMLRAS in the low-frequency range is shown in Figure 2a, where the pink shaded area is the bandgap in the calculated frequency range. For ease of discussion and analysis, a detailed schematic of the first bandgap is shown in Figure 2b. Clearly, there are a total of five complete bandgaps in the 0–500 Hz range. These five bandgaps are located between the third and fourth dispersion curves, between the sixth and seventh dispersion curves, between the eighth and ninth dispersion curves, between the tenth and eleventh dispersion curves, and between the twelfth and thirteenth dispersion curves respectively. The corresponding frequency ranges are [184.42, 184.69] Hz, [237.18, 249.80] Hz, [252.98, 272.13] Hz, [339.70, 368.00] Hz, and [368.60, 425.75] Hz respectively. For single-phase cellular metamaterials, this bandgap result is very promising and shows that lightweight SHMLRAS has excellent elastic wave attenuation properties in the low-frequency region as well as favorable prospects for engineering applications.

**Figure 3:**
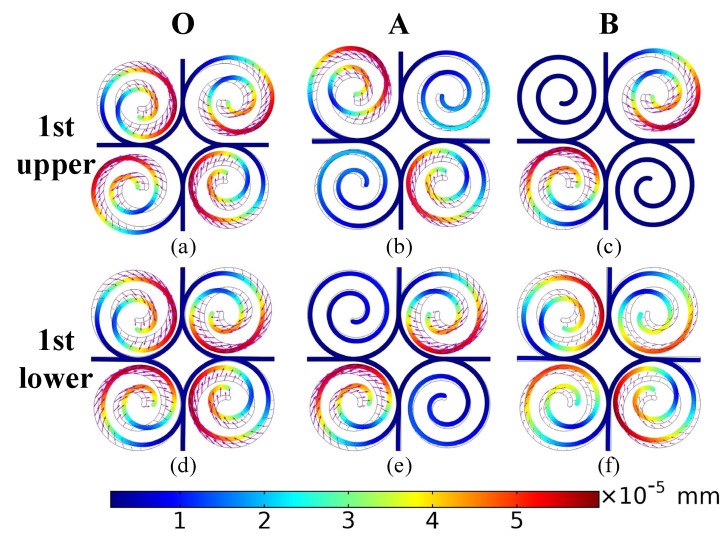
Mode shapes of the proposed structure: (**a**–**c**) represent the points (O, A, B) of the IBZ on the fourth branches the lower edge of the first bandgap; (**d**–**f**) represent the points (O, A, B) of the IBZ on the third branches the upper edge of the first bandgap; the arrows represent the magnitude and direction of the displacement of the mode. (For interpretation of the references to color in this figure legend, the reader is referred to the web version of this article.).

The application of point group theory, which is used extensively in physics and molecular chemistry, to analyze and predict the electromagnetic response of complex structures is a highly rewarding endeavor [39]. However, the mode analysis approach used in these works [38,40] more easily helps us to understand the mechanism of bandgap generation. The vibrational modes at Points O, A, and B are analyzed below for the upper (green circles) and lower (red circles) dispersion curves corresponding to the former three bandgaps in Figure 2a. The size and direction of the arrows in the mode shape diagrams represent the displacement distance and direction of the structure relative to its original position. Figure 3 shows the mode shapes at Points O, A, and B for the upper and lower boundaries of the first bandgap respectively. It can be seen that the displacement and direction of the mode shapes at the upper and lower boundaries of the fourth (1st upper) and third (1st lower) dispersion curves corresponding to the Point O are very similar in the second and fourth quadrants. In the first and third quadrants, however, the directions of the modes are opposite. At Point A, we can notice a clear exchange of energy in the structural vibrations, with the spiral vibrations in the second and fourth quadrants becoming the spiral vibrations in the first and third quadrants. In the A→B direction, attenuation occurs in the vibration modes corresponding to the fourth and third dispersion curves. Moreover, the 1st up vibration in the second and fourth quadrants decays significantly, which leads to the spiral vibration in the first three quadrants. Comparing Figure 2b with the previous discussion, we can see that the energy exchange occurring at Point A is the main reason for the creation of the first bandgap.

**Figure 4:**
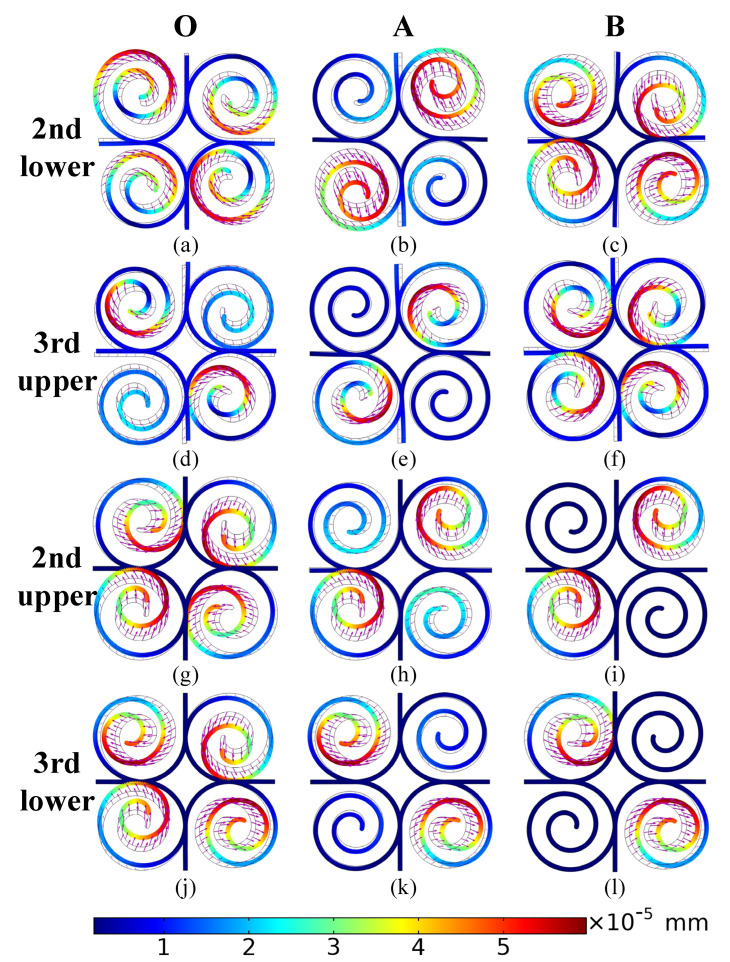
Mode shapes of the proposed structure: (**a**–**c**) represent the points (O, A, B) of the IBZ on the sixth branches the lower edge of the second bandgap; (**d**–**f**) represent the points (O, A, B) of the IBZ on the ninth branches the upper edge of the third bandgap; (**g**–**i**) represent the points (O, A, B) of the IBZ on the seventh branches the upper edge of the second bandgap; (**j**–**l**) represent the points (O, A, B) of the IBZ on the eighth branches the lower edge of the third bandgap; the arrows represent the magnitude and direction of the displacement of the mode.

Figure 4a–f shows the mode shape diagrams corresponding to the sixth (2nd lower) and ninth (3rd upper) dispersion branches at the Points O, A, and B. The marked arrows show that there is a clear difference in the direction of the spiral vibrations in the different quadrants, i.e., a greater degree of local resonance. For example, at Point B, the sixth dispersion branch tends to vibrate downwards in the first quadrant, leftwards in the second quadrant, upwards in the third quadrant, and to the right in the fourth quadrant. The ninth dispersion branch tends to vibrate in the lower left, lower right, upper right, and upper left in the four quadrants respectively. The effect of the elastic wave on the structure is weakened or completely counteracted by the effect of the local resonance spirals on the structure, i.e., there is a positive correlation between the local resonance stratification of the spiral and the attenuation performance of the elastic wave.

The local resonant mode of the unit structure is excited as the frequency approaches the natural frequency of the resonant. At this point, the elastic waves in the structure will be strongly coupled with the structural local resonant mode. Furthermore, the energy is localized due to the constant exchange into the resonant unit and the elastic wave will not propagate further. In the band structure, this is manifested by the truncation of the energy band starting at Point O by the resonant straight band, resulting in the formation of the third and fourth bandgaps. The vibration of the spirals on the seventh (2nd upper) dispersion branch is essentially constant in the third quadrant, as shown in Figure 4g–i. In the fourth quadrant, however, only the direction of vibration does not change. Meanwhile, the direction of vibration of the spiral in the second quadrant shows a similar attenuation of amplitude after reversal as in the third and fourth quadrants. On the O→A→B direction, the eighth (3rd lower) dispersion branch shows marked attenuation of the spiral amplitude in the first three quadrants. The spiral vibration only shows a reversal of direction in the second quadrant, except in the fourth quadrant which remains essentially unchanged.

Comparing Figure 3 and Figure 4, the consumption of elastic wave energy by the structure in the calculated frequency range originates from the local resonance of the spiral. Furthermore, the degree of local resonance of the spiral increases, the better performance of designed structure for elastic wave attenuation.

### 3.2. Dispersion Surfaces

In this paper, the dispersion surfaces of the SHAMLRAS periodic structure are calculated using the finite element method in the solid mechanics interface of COMSOL. The four edges of the square honeycomb metamaterials cell are divided by taking mesh = 40, i.e., the four edges of the first Brillouin zone in Figure 1d are divided into 1600 points. Once we have swept the wave vector k for all the points inside and on the boundary of the first Brillouin zone, the complete eigenfrequency surface ω=ω(k) corresponding to the different dispersion branches can be calculated using COMSOL. In this case, a complete eigenfrequency surface ω=ω(k) is also a mode. The corresponding dispersion surfaces of the different dispersion branches represent a class of modes at the corresponding eigenfrequencies. Hence, the *n*-th (n=1,2,3,…) dispersion surface can also be called the *n*-th (n=1,2,3,…) mode. Here we select modes near the first three bandgaps (third to ninth dispersion branches) for further discussion and analysis.

**Figure 5:**
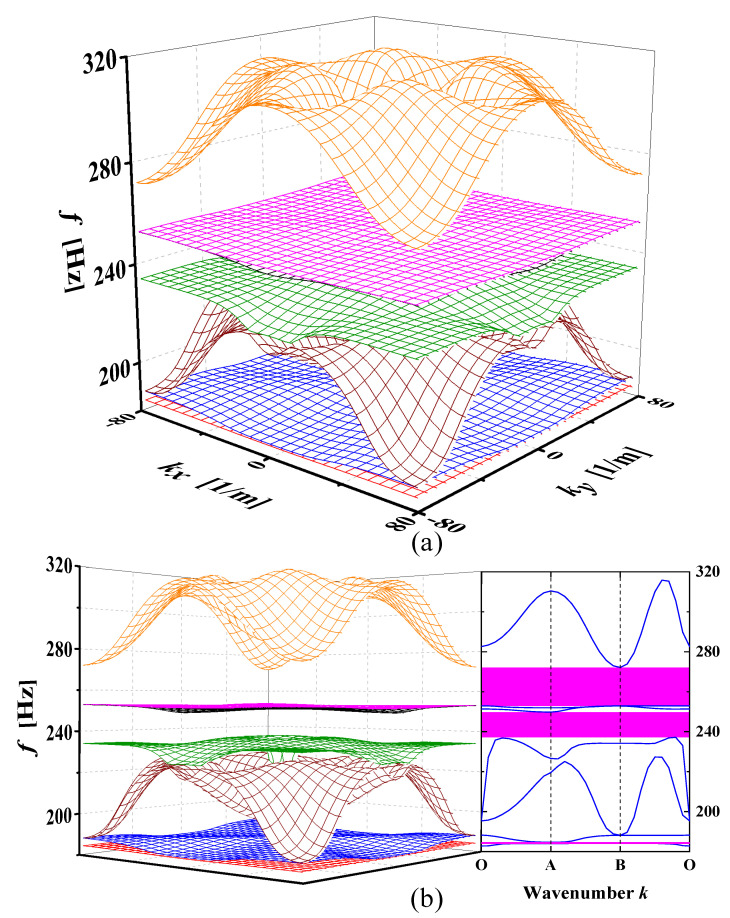
(**a**) Dispersion relations for SHAMLRAS structures in isometric perspective; (**b**) comparison of dispersion relations and band structure in horizontal view.

The dispersion surface method can be used to show the elastic wave propagation properties in the periodic structure in a three-dimensional and visual way. Figure 5a shows the dispersion relations of the seven dispersion surfaces of the third to ninth dispersion branches of the SHAMLRAS periodic structure in three-dimensional space, where the *x*- and *y*-axis coordinates represent the coordinate of the first Brillouin zone point and the z-axis represents the frequency *f*. The comparative relationship between the SHAMLRAS dispersion relation and the bandgap structure in the horizontal view is shown in Figure 5b. There is a good correspondence between the dispersion curves of the SHAMLRAS structure and the dispersion curves of the bandgap structure, as well as between the bandgap positions of the two diagrams. Simultaneously, it proves the correctness of the dispersion relation calculated by the above method.

### 3.3. Directional Propagation Property of Elastic Waves

We can obtain the ISO-frequency contours corresponding to the modes by projecting the dispersion surfaces in the $k_{x}$ and $k_{y}$ plane in Figure 5a and extracting the eigenfrequencies according to different division intervals.

The group velocity of two-dimensional periodic structures along the *x*- and *y*-directions for a given frequency can be written as:

(13) $$c_{gx}=a_{x}\frac{\partial \omega }{\partial k_{x}},\;c_{gy}=a_{y}\frac{\partial \omega }{\partial k_{y}}$$

where $a_{x}$ and $a_{y}$ denote the lattice constants in the *x*- and *y*-directions respectively.

The group velocity is defined as the gradient of an ISO-frequency curve when the dispersion surface is described by a two-dimensional contour. At the same time, the direction of the outer normal at each point on the contour is the direction of the group velocity at that point, which represents the direction of energy propagation of the vibration at that frequency. Therefore, the group velocity can be used to express the speed of energy propagation as well as the direction and magnitude of the elastic wave propagation. With the calculation of the gradient at each point on the contour, we can obtain the direction of propagation and the region of propagation for a given frequency of the vibration. The values of $c_{gx}$ and $c_{gy}$ are used as the *x*- and *y*-coordinates to obtain the group velocity for a specific frequency.

**Figure 6:**
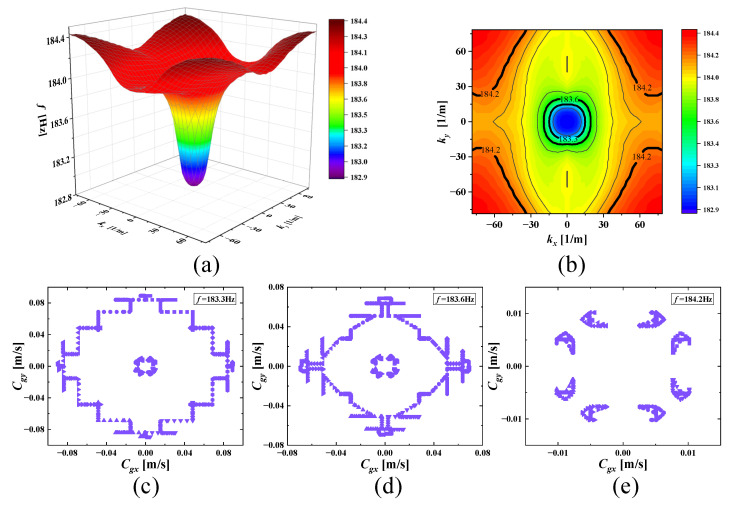
(**a**) Third mode; (**b**) ISO-frequency contours (the thick black solid lines are the three selected frequencies to be analyzed and similar depictions can be seen in Figure 7, Figure 8, Figure 9, Figure 10, Figure 11 and Figure 12); (**c**–**e**) group velocity of third wave mode at 183.3 Hz, 183.6 Hz, and 184.2 Hz.

**Figure 7:**
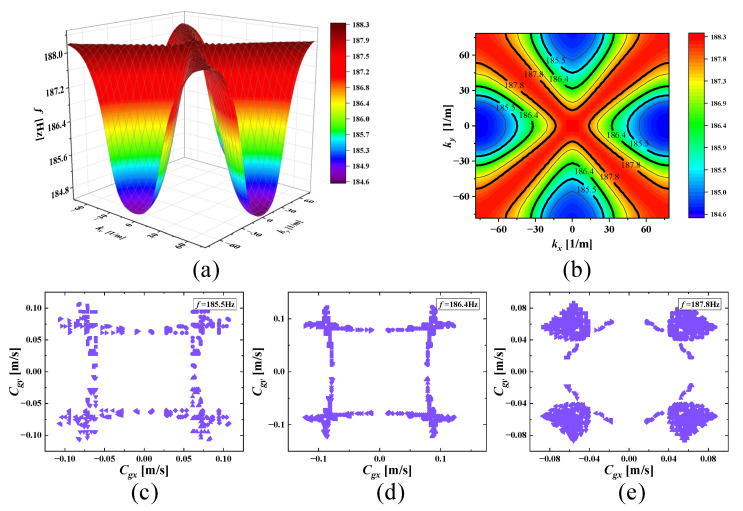
(**a**) Fourth mode; (**b**) ISO-frequency contours; (**c**–**e**) group velocity of fourth wave mode at 185.5 Hz, 186.4 Hz, and 187.8 Hz.

**Figure 8:**
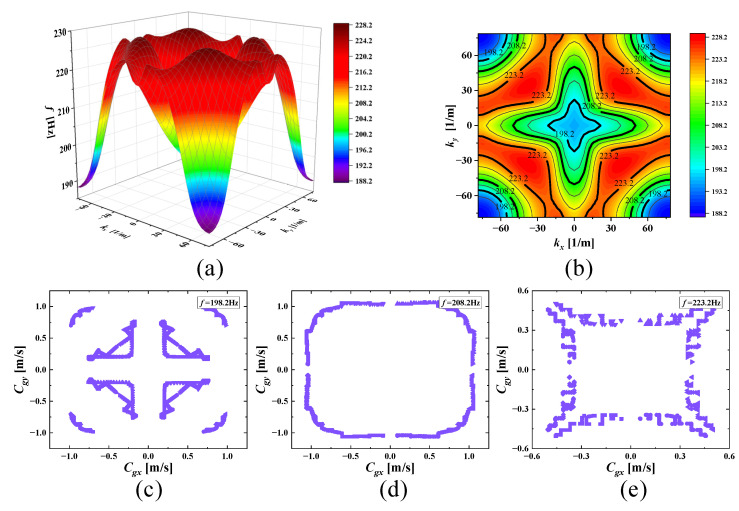
(**a**) Fifth mode; (**b**) ISO-frequency contours; (**c**–**e**) group velocity of fifth wave mode at 198.2 Hz, 208.2 Hz, and 223.2 Hz.

**Figure 9:**
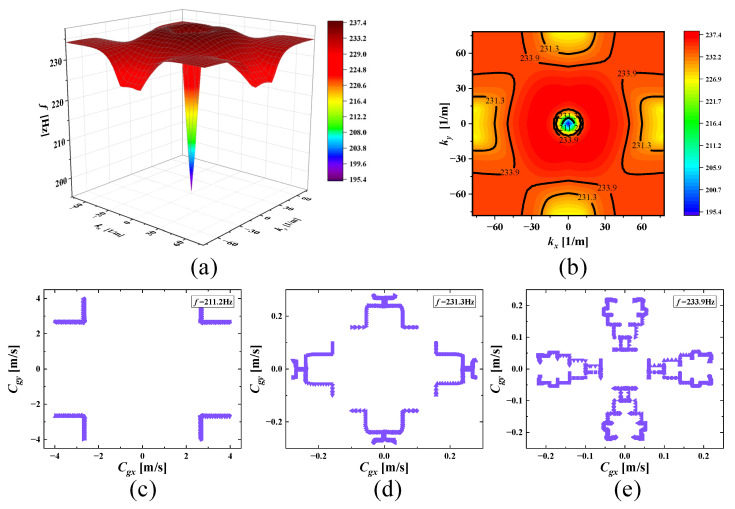
(**a**) Sixth mode; (**b**) ISO-frequency contours; (**c**–**e**) group velocity of sixth wave mode at 211.2 Hz, 231.3 Hz, and 233.9 Hz.

**Figure 10:**
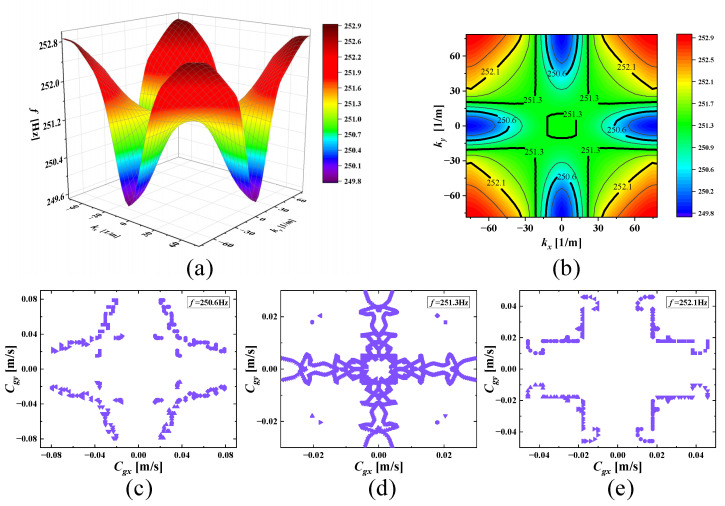
(**a**) Seventh mode; (**b**) ISO-frequency contours; (**c**–**e**) group velocity of seventh wave mode at 250.6 Hz, 251.3 Hz, and 252.1 Hz.

**Figure 11:**
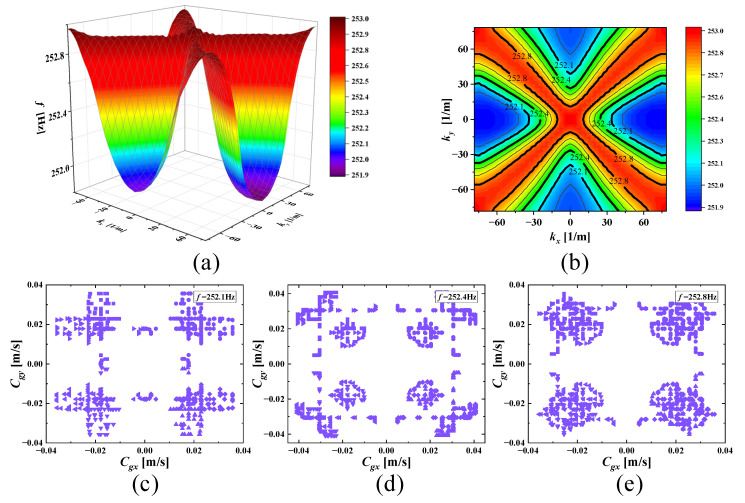
(**a**) Eighth mode; (**b**) ISO-frequency contours; (**c**–**e**) group velocity of eighth wave mode at 252.1 Hz, 252.4 Hz, and 252.8 Hz.

**Figure 12:**
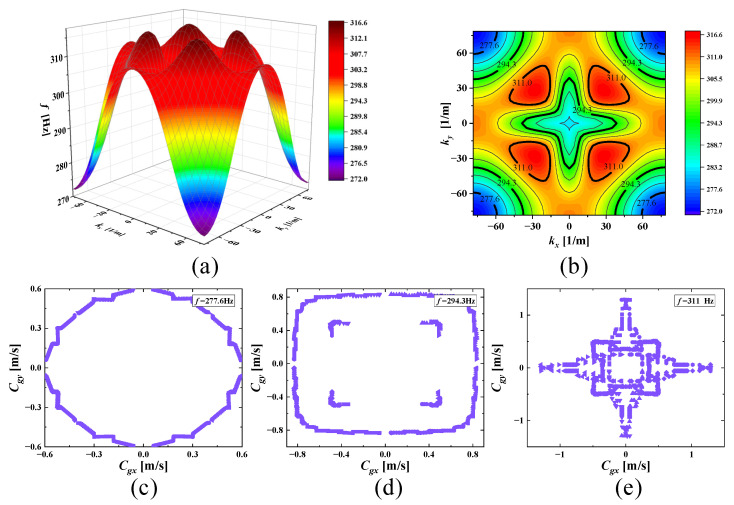
(**a**) Ninth mode; (**b**) ISO-frequency contours; (**c**–**e**) group velocity of ninth wave mode at 277.6 Hz, 294.3 Hz, and 311 Hz.

With increasing frequencies, the SHAMLRAS structure vibration propagation is mostly concentrated in the $k_{x}$ and $k_{y}$ directions, as shown in Figure 6, Figure 7, Figure 8, Figure 9, Figure 10, Figure 11 and Figure 12. At the same time, the vibration propagation generally behaves as follows: it is enhanced, then weakened, and then enhanced again. In particular, the sixth mode case has the weakest vibration propagation in the $k_{x}$ and $k_{y}$ directions. On the other hand, we can observe from Figure 6a, Figure 7a, Figure 8a, Figure 9a, Figure 10a, Figure 11a and Figure 12a that there is weak anisotropy in the $k_{x}$ and $k_{y}$ directions for the fifth and sixth modes at the lower frequencies of the dispersive surface, whereas there are strong anisotropy in this direction for the fourth, seventh and eighth modes. Furthermore, only the fifth and ninth modes have strong anisotropy in the direction with the diagonal as the axis of symmetry.

To be more precise in evaluating the propagation of elastic waves on different dispersion surfaces, the ISO-frequency contours for the three frequency cases are marked in (b) of Figure 6, Figure 7, Figure 8, Figure 9, Figure 10, Figure 11 and Figure 12 with heavy black solid lines, and the group velocities corresponding to these ISO-frequency contours are shown in (c,d) of Figure 6, Figure 7, Figure 8, Figure 9, Figure 10, Figure 11 and Figure 12 respectively. The group velocity in Figure 6c–e shows a distribution of points in the 0° to 360° direction. The points on the outside of the group velocity are more densely distributed in the $k_{x}$ and $k_{y}$ directions as the frequency increases, however, the group velocity points in this direction disappear with the frequency increases to *f* = 184.2 Hz. In contrast, with increasing frequency *f* the group velocity distribution is more densely distributed in the region near the diagonal as the axis of symmetry.

The clusters of velocities in the $k_{x}$ and $k_{y}$ directions increase remarkably with increasing frequency, and the points in the other directions decrease significantly, according to (c,d) of Figure 9, Figure 10 and Figure 12. However, a highly visible concentration of group velocity points in the diagonal direction of the graph is observed in Figure 6c,d as well as in Figure 11c,d when the frequencies are located in the fourth and eighth modes, and the degree of the clusters increases noticeably with increasing frequency. That demonstrates that there is a strong energy aggregation at this location, and it also indicates that the propagation of energy in the $k_{x}$ and $k_{y}$ direction is much weaker in this direction compared to the diagonal direction due to the small group velocity distribution. Consequently, we can consider that elastic waves form vibration blind zones in the $k_{x}$ and $k_{y}$ directions, and this characteristic is very useful for vibration isolation needs in specific directions of engineering.

## 4. Influence of the SHAMLRAS Bandgap

### 4.1. Influence of Spiral Geometry on the Bandgap

From the analysis in Section 2.1, the structure of SHAMLRAS changes markedly when the inner diameter (*R*_2_), turn number (*n*), circle distance (*d*), the tart parameters (*t*_0_), and the End parameters (*t*_1_) of parameter *t* are changed in Equation (5). The changes in these parameters are further influenced by the bandgap characteristics of the SHAMLRAS structure and the dispersion relations. To be more flexible in dealing with different frequencies of elastic waves in engineering damping applications, the influence of the spiral geometry parameters on the bandgap width and position is discussed below.

**Figure 13:**
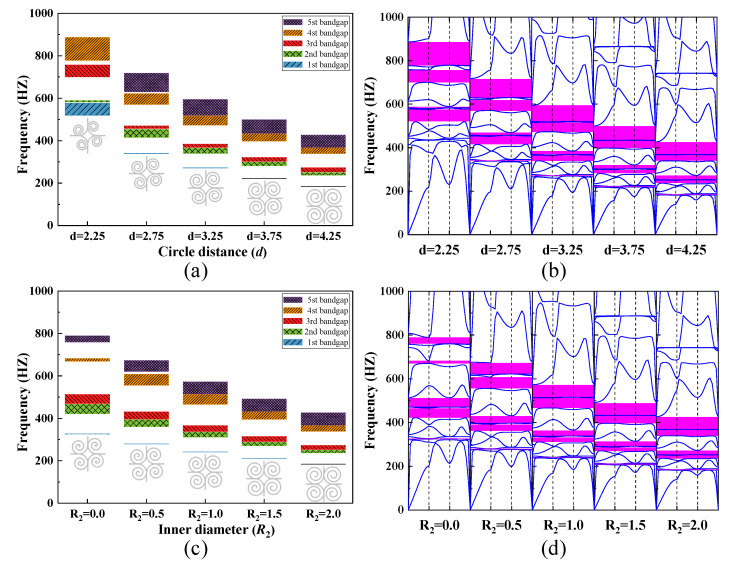
The dependence of the bandgap width on (**a**,**b**) the circle distance *d*, and (**c**,**d**) the inner diameter *R*_2_.

The influence of the circle distance *d* and the inner diameter *R*_2_ on the bandgap width of the SHAMLRAS structure are shown in Figure 13a–d respectively. With increasing circle distance *d* in the range of 1000 Hz, the second bandgap width is found to show an increase followed by a decrease. Although the bandgap width of the SHAMLRAS structure decreases as the circle distance *d* increases, the frequency of each bandgap also decreases remarkably. Furthermore, a comparison of Figure 13a,b shows that the first bandgap is generated between the third and fourth dispersion curves when *d* = 2.75, 3.25, 3.75, 4.25. The second to fifth bandgaps then corresponds to between the fifth and sixth dispersion curves, between the seventh and eighth dispersion curves, between the ninth and tenth dispersion curves, and between the eleventh and twelfth dispersion curves respectively. However, in the case of *d* = 2.25, no bandgap can be observed to be created between the third and fourth dispersion curves, with the first bandgap located between the sixth and seventh dispersion curves. As the inner diameter *R*_2_ is varied, the width of the first three bandgaps is in Figure 13c is negatively correlated with the change in *R*_2_, while only the width of the fifth bandgap is positively correlated with the change in *R*_2_, with only the fourth bandgap showing an increase followed by a decrease. Moreover, the position of the dispersion curves for the first five bandgaps of the SHAMLRAS structure has not changed by comparing Figure 13a,b. From the above discussion, there is a clear indication that we should increase the value of the circle distance *d* as well as *R*_2_ in the SHAMLRAS structure within a limited dimension when we need a low-frequency bandgap.

**Figure 14:**
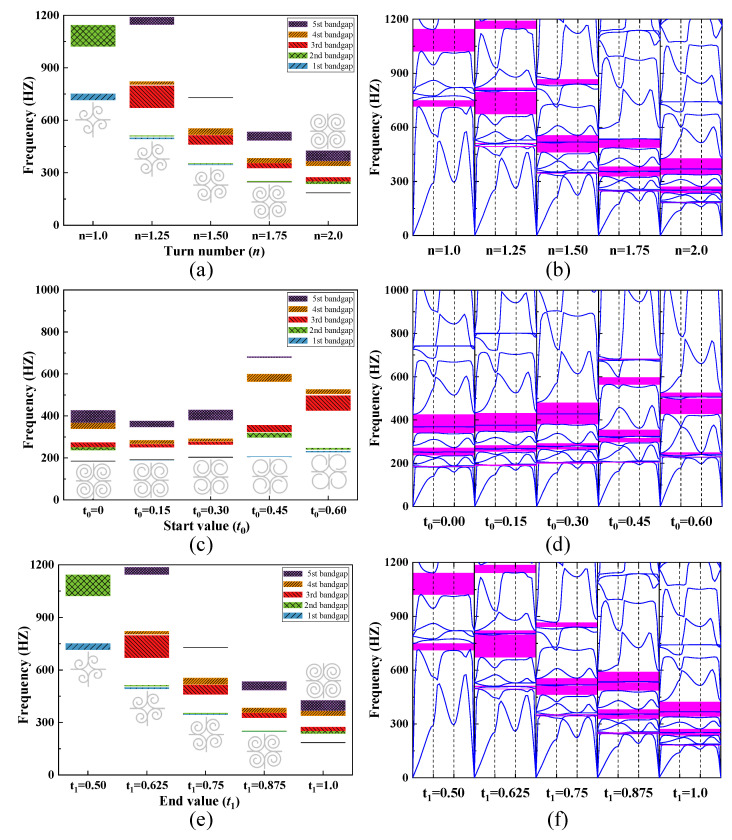
The dependence of the bandgap width on (**a**,**b**) the turn number *n*, (**c**,**d**) the start value *t*_0_, and (**e**,**f**) the end value *t*_1_.

The dependence of the bandgap width on turn number *n*, starting value *t*_0_, and end value *t*_1_ is shown in Figure 14a–f respectively. All the shapes of the SHAMLRAS structures are noted to change more markedly with the variation of parameters. The variation of these parameters also leads to changes in the position of the bandgap of the SHAMLRAS structure between the different dispersion curves, especially in the low-frequency bandgap region. There is a narrower bandgap that is consistent between the second and third dispersion curves as the values of these three parameters gradually approach the structural parameters selected in Table 2. As the selected parameter change to the structural parameters shown in Table 2, the bandgap tends to decrease at all frequency positions, in particular, the frequency values between the second and third dispersion curves overlap, and the bandgap at this position disappears. We also observe an interesting phenomenon: there is a striking similarity in the effect of the turn number *n* in Figure 14a,b and the end value *t*_1_ in Figure 14e,f on the SHAMLRAS geometry and band structure. This suggests a strong one-to-one correspondence between the turn number *n* and the end value *t*_1_ for the SHAMLRAS structure. On the other hand, variations of the three parameters in Figure 14 have a relatively high effect on the bandgap width of the SHAMLRAS structure. Although the variation in bandgap width at this point does not have the same continuity of variation as the bandgap width shown in Figure 13, the position of the dispersion curve is the same for each bandgap, except for the structural parameters in Table 2.

In the above case, the dispersion curves of the second to fifth bandgaps of the different SHAMLRAS structures are located between the third and fourth dispersion curves, between the sixth and seventh dispersion curves, between the eighth and ninth dispersion curves, and between the tenth and eleventh dispersion curves respectively. This indicates that the relationship between the position of the bandgap and dispersion curves is relatively stable for the range of parameters chosen.

### 4.2. Influence of Spiral Arrangement on the Bandgap

**Figure 15:**
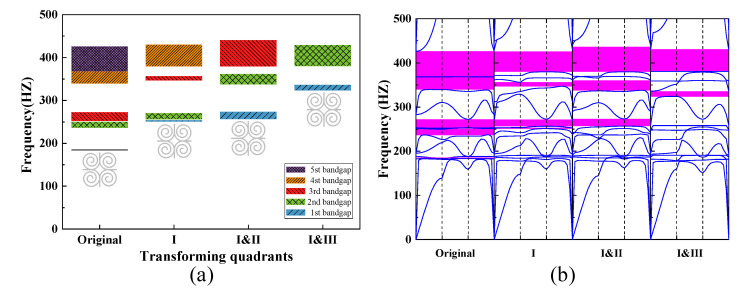
The dependence of the bandgap width on the spiral (**a**,**b**) transforming quadrants, where the number “original” represents the bandgap distribution for the parameter shown in Table 2, the number “I” represents a transformation of the spiral arrangement in the first quadrant, the numbers “I&II” represents a transformation of the spiral arrangement in the first and second quadrants, and the numbers “I&III” represents a transformation of the spiral arrangement in the first and third quadrants.

The effect on bandgap width and position of spirals in a square honeycomb structure is explored here. As shown in Figure 15, the bandgap width distribution is calculated for each case by varying the arrangement of the spirals in different quadrants. When the spiral arrangement is varied in accordance with Figure 15, there are decreases to disappear of the bandgap between the third and fourth dispersion curves, the bandgap between the sixth and seventh dispersion curves, and the bandgap between the eighth and ninth dispersion curves when the spiral arrangement is transformed in the first and third quadrants. The different arrangement of spirals demonstrates that only the first three bandgaps of structural parameters shown in Table 2 are more significantly affected.

### 4.3. Influence of Material Parameters on the Bandgap

**Figure 16:**
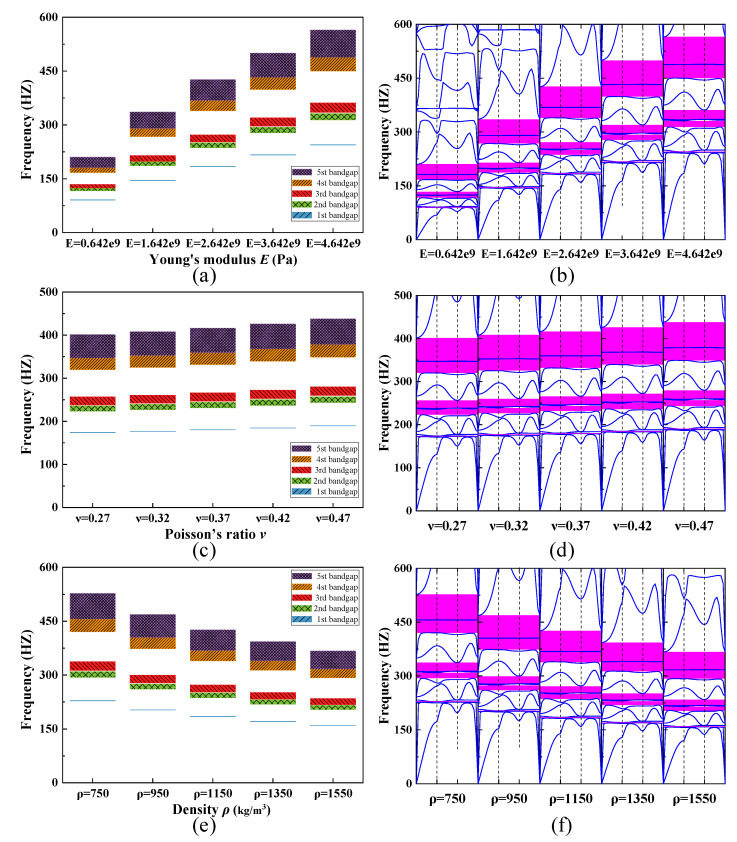
The dependence of the bandgap width on (**a**,**b**) Young’s modulus *E*, (**c**,**d**) the Poisson’s ratio *ν*, and (**e**,**f**) the density *ρ*.

The influence of the material parameters Young’s modulus *E*, Poisson’s ratio *ν*, and density *ρ* (only one of these parameters is changed at a time) on the width and position of the bandgap in the numerical simulation are illustrated in Figure 16. The frequency position of each bandgap rises gradually with increasing Young’s modulus *E* and Poisson’s ratio *ν* as shown in Figure 16a,c. Simultaneously, the width of the five bandgaps shown in the diagram increases. In contrast, both the width and position of the bandgap in Figure 16e show a negative correlation with the variation in density *ρ*. Additionally, it can be observed that higher frequency bandgaps are more sensitive to the change of material parameters. In other words, the higher the bandgap frequency, the faster the bandgap width increases (or decreases) with the change of material parameters. On the other hand, the relative positions of each bandgap have not changed in the dispersion curves. It is demonstrated that changes in Young’s modulus *E*, Poisson’s ratio *ν,* and density *ρ* during the simulations do not open a new bandgap (or disappear the existing bandgap) in the band structure of SHAMLRAS. As the analysis above shows, the smaller the values of Young’s modulus *E* and Poisson’s ratio *ρ* of the material, the lower the frequency of the bandgap. At the same time, it is easier to obtain bandgaps at lower frequencies with higher values of the density *ρ*.

## 5. Filtering Properties of the Finite Periodic Structure of the SHAMLRAS

**Figure 17:**
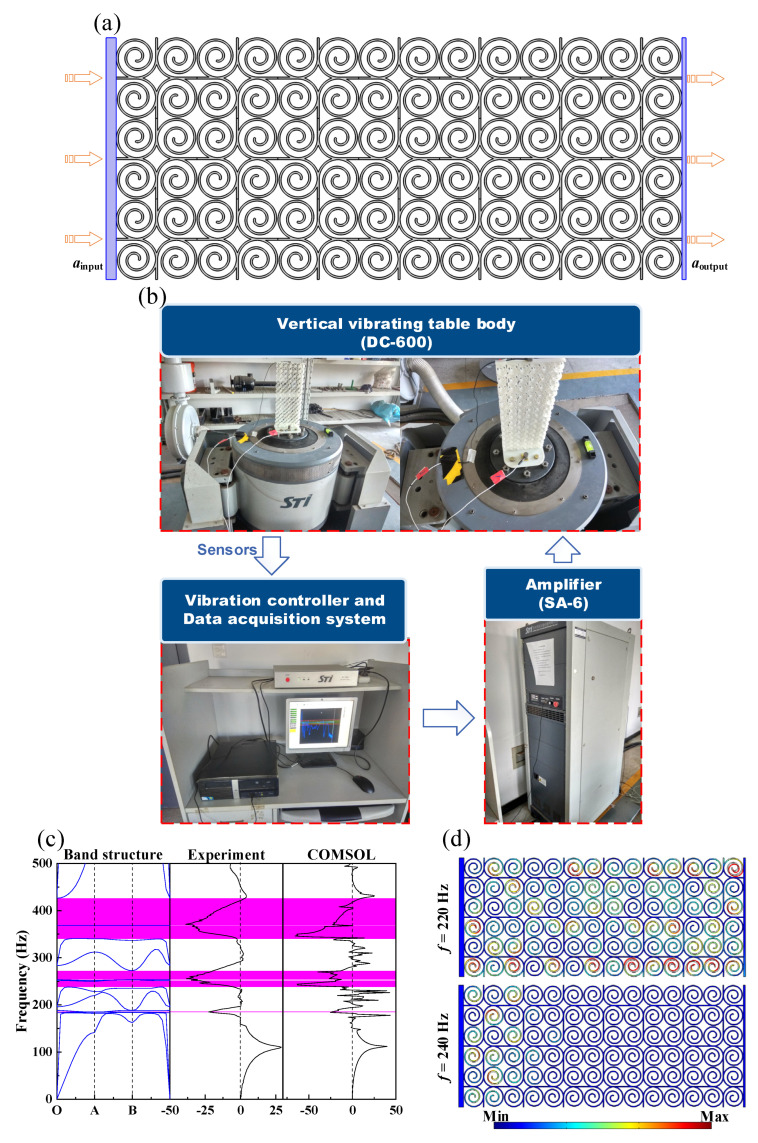
(**a**) Geometry and loading conditions of the SHAMLRAS periodic structure; (**b**) experimental setup for transmission loss in SHAMLRAS periodic structures; (**c**) comparative spectrum diagram obtained from transmission loss experiment and simulation; (**d**) deformation of SHAMLRAS periodic structures under harmonic loading frequency at *f* = 220 Hz (outside the bandgap region) and *f* = 240 Hz (inside the bandgap region).

The dispersion relations and free wave propagation properties in the case of SHAMLRAS infinity structures have been analyzed in the previous sections, but they may not be adaptable to the demands of flexible load-bearing in engineering. In this section, the performance of SHAMLRAS for vibration isolation finite structures is investigated from both experimental tests and numerical simulations. The geometry and loading environment in Figure 17a were used for both the transmission loss experiments and the COMSOL simulations. To evaluate the vibration isolation performance of the SHAMLRAS structure, the frequency response function (FRF) was calculated with the left-hand panels of 5 mm thickness as the excitation input and the right-hand panels of 2 mm thickness as the response receiver. The geometric and material parameters shown in Table 2 were used as the basis for obtaining the specimens required in the experiments through 3D printing, and the experiment was carried out based on the transmission loss experimental setup shown in Figure 17b. After the printed specimen has been fixed to the vertical vibration table through the 5 mm thick prefabricated base, the excitation and response data from the experimental test are calculated using an accelerometer to obtain the spectrum in Figure 17c. We used acceleration as the input signal for excitation in both experimental and simulations, and the calculated spectra were placed in Figure 17c for comparative analysis.

Five decay peaks are corresponding to the band structure that we observed both in experimental and in COMSOL simulations, and their frequencies and ranges match well with the calculated bandgaps in Figure 17c. At the same time, the spectrogram shows a significant amplification at approximately 120 Hz. This amplification phenomenon is attributed to the resonance in this frequency region of the SHAMLRAS periodic structure used in the experiments and simulations. It is shown that as the sweep frequency increases, the vibration frequency of the vertical vibrating table gradually coincides with the eigenfrequency of the SHAMLRAS periodic structure. At this point, the structure deformed significantly and is represented by a resonance peak in the spectrogram.

In addition, the behavior of the elastic wave propagation is shown in Figure 17d for excitation frequencies of *f* = 220 Hz (outside the bandgap range) as well as for frequencies *f* = 240 Hz (inside the bandgap range). The elastic wave is significantly suppressed when the excitation frequency *f* = 240 Hz is in the bandgap range, which corresponds well to the bandgap shown in Figure 2a.

## 6. Conclusions

In this paper, lightweight single-phase acoustic metamaterials with low-frequency bandgap properties are designed by combining a square honeycomb structure with multiple Archimedean spirals.

The existent Archimedean spirals are shown to open multiple complete bandgaps below 500 Hz with Bloch’s theorem and finite element analysis. The vibrational modes are discussed for the dispersion curves near the first three bandgaps at the boundary points of the IBZ. The generation of low-frequency bandgaps was found to be related to the degree of local resonance of spirals. Characteristics of the directional propagation of elastic waves in SHAMLRAS periodic structures and the attenuation properties of in-plane vibrations are analyzed using dispersion surfaces and group velocities at specific frequencies. Optimizing the width and position of the low-frequency bandgap to enhance wave attenuation can be achieved by adjusting the material parameters, the spiral arrangement, and the parameters of the control equations. In this case, the spirals in circular arrays, the increase of the circle distance *d* and the inner diameter *R*_2_ within a limited dimension are helpful in obtaining a lower frequency bandgap. In the end, the spectrum obtained through transmission loss experiments and COMSOL simulations are used to demonstrate and verify the vibration isolation performance of SHAMLRAS with limited dimensions. That proves the great potential of the SHAMLRAS structure in achieving low-frequency noise and vibration control using single-phase materials.

## Figures and Tables

**Table 1:** Coordinates of the boundary points of the IBZ.

Boundary Points	Cartesian Basis	Reciprocal Basis
O	(0, 0)	(0, 0)
A	(π/L, 0)	(1, 0)
B	(π/L, π/L)	(1, 1)

**Table 2:** Geometry and material parameters of the structure.

Lattice Parameters		Material Parameters of Photosensitive Resin	
Radius of tangent circle	*R*_1_ = 9.5 mm	Young’s modulus	2.642 GPa
Inner diameter	*R*_2_ = 2.0 mm	Density	1150 kg/m^3^
Circle distance	*d* = 4.25 mm	Poisson’s ratio	0.42
Ligament thickness	*p* = 1.0 mm		
Turn number	*n* = 2		
Start value	*t*_0_ = 0		
End value	*t*_1_ = 1		

## Data Availability

Data are contained within the article.
